# Solution-Route Inkjet Fabrication of CeO_2_ Thin Films with Tunable Microstructure

**DOI:** 10.3390/ma16041685

**Published:** 2023-02-17

**Authors:** Veena Singh, Lyubov Belova

**Affiliations:** Department of Material Science and Engineering, KTH-Royal Institute of Technology, SE-100 44 Stockholm, Sweden

**Keywords:** CeO_2_, inkjet printing, microstructure, thin films

## Abstract

We report the fabrication and characterization of solution-route CeO_2_ thin films with a tunable porosity and microstructure. Films were deposited by means of inkjet printing technique using 0.2 M, 0.4 M and 0.6 M concentration inks prepared from Ce(NO_3_)_3_·6H_2_O precursor. Printing was performed at two different temperatures of 60 °C and 300 °C to study the variation in structure. Printing parameters were adjusted for the consecutive deposition of layers, resulting in ≈140 nm and ≈185 nm thick single layers for 60 °C and 300 °C printing temperatures, respectively. We compared the microstructure of printed films for different concentrations, printing temperatures, solvents and substrates. The formation of the cubic fluorite structure of the printed films was confirmed via XRD characterization. We suggest this technique as an advanced method for high-quality film fabrication with a controlled microstructure and with a minimal waste of materials. Through adjusting printing parameters, both dense and porous films can be produced for use in different applications.

## 1. Introduction

Ultra-porous structured oxides have an extensive potential for a wide range of applications, including chemical sensors, catalysis as well as fuel cells, resulting from their gas permeability, higher surface/volume ratio and unique chemical properties [1]. Among metal oxides, cerium oxide (CeO_2_) has more stability, redox behavior and fluorite crystalline structure (FCC) with an Fm_3_m space group. That contributes to a reactive surface area towards free radical neutralization [2]. Depending on the environment, CeO_2_ can exist in Ce^3+^ and Ce^4+^ oxidation states and has a distinctive capability to switch between oxidation states, creating oxygen vacancies in the crystal lattice [3]. CeO_2_ and ceria-based materials play a significant role as fast ion-oxide conductors and are hence the most promising materials for application in sensors [4], membrane reactors [5], fuel cells [5,6], environmental catalysis [7], etc. Furthermore, along with ionic conduction, ceria also displays a high oxygen mobility or diffusion and an excellent structural stability [8].

Prior research demonstrated that different CeO_2_ properties usually depend on preparation routes, the degree of crystallinity, and different morphologies. Thus, by tailoring the morphology, it is possible to design an “ideal” material for a particular purpose, and the resulting microstructure may have a significant influence for various CeO_2_ applications [9].

The fabrication of ceria films based on different techniques, such as metal-organic chemical vapor deposition (MOCVD) [10], pulsed-laser deposition (PLD) [11], electrodeposition [12], electrochemical liquid deposition (ELD) [13], electron-beam evaporation [14], Sol-gel spin coating [15], electro-precipitation [16] and dc-magnetron sputtering [17], has already been reported. However, these techniques had some drawbacks, including their high cost, the difficulty to produce large-area films, their material wastage, their need for a highly controlled environment, their inconsistent reproducibility, their significant energy and their time consumption. On the other hand, inkjet printing (IJP) is an alternative promising technique for designing micro to macro-scale high-quality thin films in ambient conditions. Inkjet is a low-cost, waste-free, energy-efficient direct mask-free patterning/deposition technique scalable to an industrial level. Among other advantages, this technique has a substantial value in sustainable production. The drop-on-demand approach means that the material is only deposited when and where it is required, and no material is wasted during deposition. Additive-nature and digital-patterning features eliminate material waste for eventual lithography where patterned surfaces are needed. The low energy consumption of these tools along with the absence of vacuum equipment further add to the sustainability of this fabrication approach. IJP allows for a high reproducibility for the thickness, microstructure and composition of the films, making it attractive for technological applications, including thin film transistors, photonic crystals, capacitors, fuel cells, ferroelectric or dielectric materials, etc. 

Gallage et al. [18] reported the fabrication and patterning of CeO_2_ films by inkjet deposition with and without chelating agent, where dense films of 200–350 nm thickness were obtained. In the present work, we demonstrate the possibility to tailor an inkjet-printed CeO_2_ microstructure from being dense to highly porous by modifying the printing parameters and strategies, including printing temperatures, ink concentrations, ink solvents and substrate surfaces, rendering such structures suitable for different uses. 

## 2. Materials and Methods

We used cerium (III) nitrate hexahydrate Ce(NO_3_)_3_·6H_2_O (Sigma Aldrich, St. Louis, MI, USA) as a precursor for CeO_2_ deposition. We performed thermogravimetric analysis (TGA) in the range of 50 °C to 500 °C on a Perkin Elmer TGS-2 Thermo-gravimetric analyzer (PerkinElmer, Waltham, MA, USA) to analyze the decomposition behavior of the precursor. 

Inks were designed based on their solution chemistry. Ce(NO_3_)_3_·6H_2_O was completely dissolved in 2-isopropoxy ethanol (IPE) solvent by ultrasonicating at <20 °C for 30 min. Sonication parameters were optimized to obtain a homogeneous solution and avoid the formation of micro-bubbles. We prepared inks having different precursor concentrations of 0.2 M, 0.4 M and 0.6 M. To study the microstructural variation, we also used different combinations of solvents during ink preparation with ethanol:ethylene glycol (Et:Etg), IPE with surfactant triton-X. All films were printed on glass substrates cut from standard microscope glass slides. We also studied the differences in film formation on smooth and rough (frosted) glass substrates. All the substrates, prior to printing, were immersed and sonicated in the sequence of solutions of soap, distilled water, acetone, and ethanol, followed by isopropanol, and were finally dried by an N_2_ gun. Ce(NO_3_)_3_·6H_2_O films with a geometric surface area of 0.9 cm × 1.5 cm were inkjet-printed on preheated substrates by a home-built piezoelectric DOD inkjet printer using an 80 pl Xaar (Cambridge, UK) printhead. We tested different substrate temperatures: 60 °C, 100 °C, 150 °C and 300 °C. The temperature ranges were selected to be below and above the thermal decomposition of the precursor, respectively. Among these, using 60 °C and 300 °C substrate temperatures resulted in the most homogeneous and uniform films. For other printing temperatures, we observed a poor adhesion to the substrate, flaking, as well as extensive film cracking. Hence, for further study, we used the optimized 60 °C and 300 °C substrate temperatures for inkjet printing. After printing, all the films were annealed in air at 400 °C for 45 min. 

Thicker films were obtained by repeating the printing process sequentially and obtaining multiple printed layers. All the different inkjet printing parameters used in the present work are summarized in Table 1.

A focused ion beam/scanning electron microscope (FIB/SEM, FEI Nova 600 Nanolab, Waltham, MS, USA) was used for surface and cross-sectional imaging. To confirm the crystallinity and analyze the crystallographic phases of the printed thin films, X-ray diffraction (XRD, Siemens D5000, Siemens, Munich, Germany) was performed in the range of 15° to 85° with 0.02° per step and a 3 s step time.

## 3. Results and Discussion

**Figure 1:**
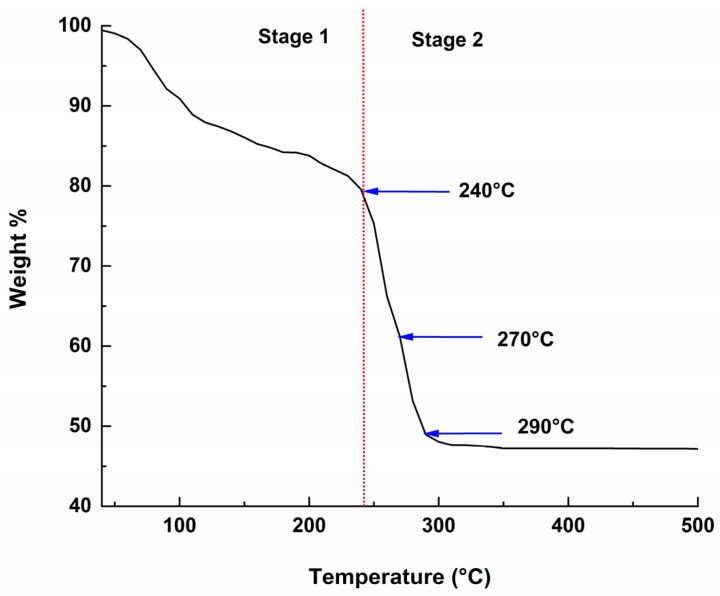
TGA curve of Ce(NO_3_)_3_·6H_2_O.

Figure 1 shows the decomposition behavior of Ce(NO_3_)_3_·6H_2_O precursor performed from 50 °C to 500 °C. The TGA curve shows that the decomposition occurred in two stages: first-stage weight loss at <240 °C is mainly due to dehydration, while nitrate decomposition causes second-stage weight loss after 240 °C to >300 °C, which results in the final cerium oxide CeO_2_ product [19].

Both ink compositions and printing parameters were then systematically varied to obtain homogeneous films with different microstructures. This included variations of: precursor concentration, solvent ratios, the use of surfactants, substrate roughness, as well as printing temperatures. After optimization of the substrate temperatures as described above, we observed that the produced films showed a diverse microstructure and porosity; hence, depending on the required application, we can tune the microstructure by adjusting the printing parameters. 

**Figure 2:**
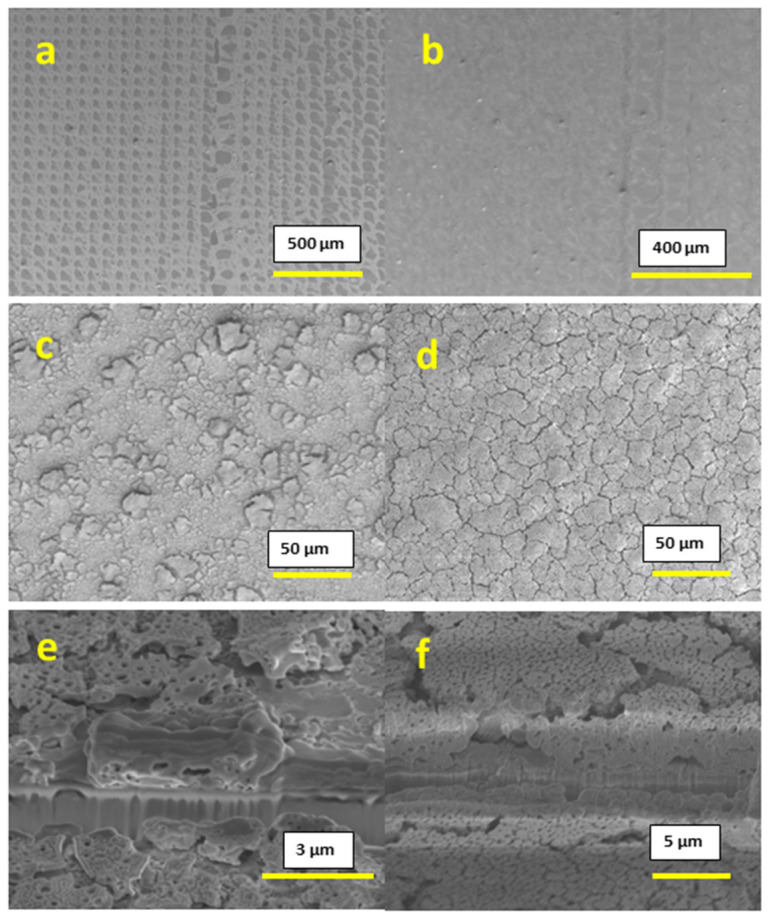
SEM images: (**a**) Surface image of 0.2 M conc. 1-layer film; (**b**) surface image of 0.2 M conc. 10-layer film; (**c**) surface image of 0.6 M conc. 1-layer film; (**d**) surface image of 0.6 M conc. 10-layer film; (**e**) cross-section of 0.6 M conc. 1-layer film; (**f**) cross-section of 0.6 M conc. 10-layer film.

The films that were printed at 300 °C with 0.2 M concentration inks displayed incomplete substrate coverage due to extremely rapid drying, combined with a small quantity of the precursor material at this concentration, as shown in Figure 2a,b; the surface of the substrate is clearly visible for both the cases of 1 printed layer and 10 printed layers, and thus this concentration is inappropriate for applications that require continuous and homogeneous films. In the case of 0.6 M concentration inks, the printed films showed complete coverage of the substrate, but part of the film had a poor adhesion towards the substrate, which resulted in flakes that peeled off from the surface, as shown in Figure 2c,d. This peeling was observed for both the cases of 1-layer and 10-layer films. Cross-section images for 0.6 M concentration films support this conclusion and are shown in Figure 2e,f. 

**Figure 3:**
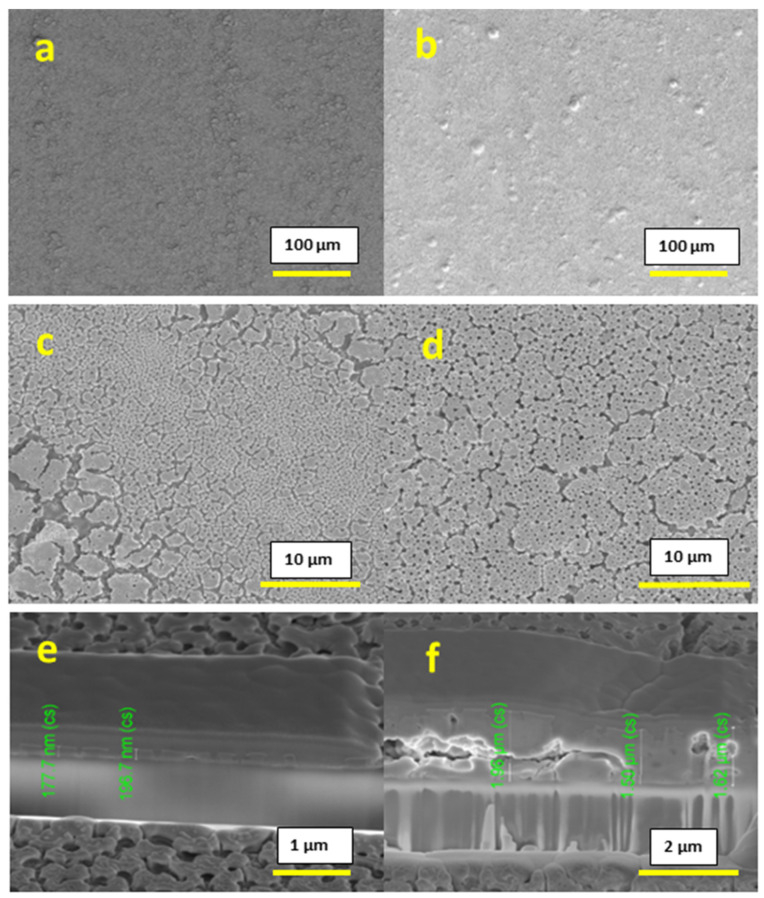
SEM images: (**a**,**c**) surface image of 0.4 M conc. 1-layer film; (**b**,**d**) surface image of 0.4 M conc. 10-layer film; (**e**) cross-section of 0.4 M conc. 1-layer film; (**f**) cross-section of 0.6 M conc. 10-layer film.

After printing the 0.4 M concentration inks, we observed a uniform and homogeneous surface without any peeling for both 1-layer and 10-layer films, as shown in Figure 3a–d. The thickness obtained was 177–196 nm for the 1-layer film and 1.60–1.96 µm for the 10-layer film, as shown in Figure 3e,f respectively. Both surface imaging and cross-section analysis showed the presence of a substantial amount of pores throughout the film.

Due to the observed homogeneity of the films, for further study, we considered a 0.4 M concentration as the optimum concentration.

**Figure 4:**
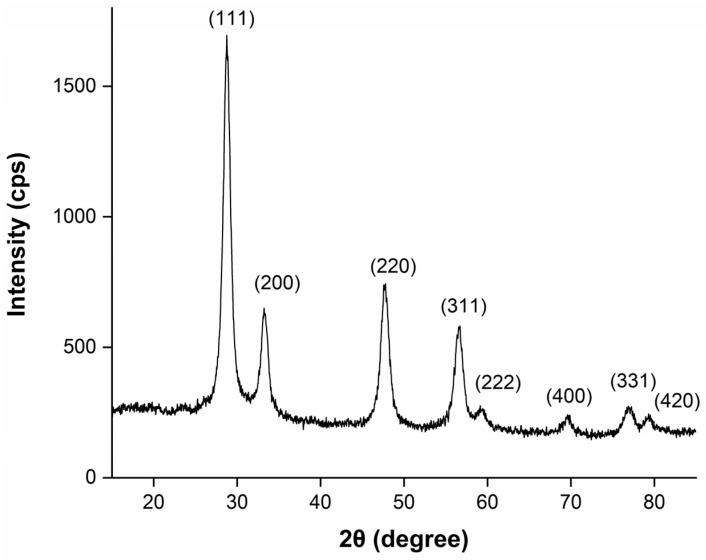
XRD of a 10-layer inkjet-printed CeO_2_ film from 0.4 M ink.

The crystallinity and crystallographic phase identification for the printed thin film was conducted by using X-ray diffraction (XRD). Figure 4 shows the XRD pattern of a CeO_2_ film obtained from a 0.4 M conc. 10-layer inkjet-printed film on a glass substrate.

Diffraction peaks observed at 28.66°, 33.22°, 47.69°, 56.59°, 59.36°, 69.74°, 77.08° and 79.47° corresponded to (111), (200), (220), (311), (222), (400), (331) and (420), respectively. From the XRD diffraction peaks, it is clear that CeO_2_ peaks are in good agreement with the cubic fluorite crystalline structure (ICSD ref. code 01-075-0076), with a lattice constant of a = b = c = 5.3890 Å. No extra peaks were observed from the measurement. 

**Figure 5:**
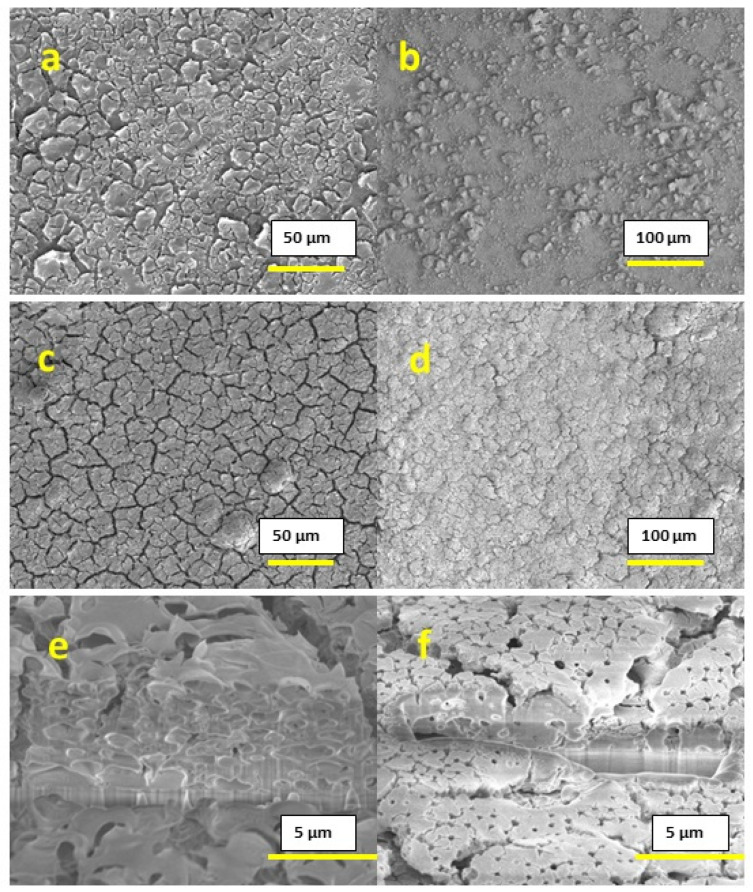
SEM images of films based on 0.4 M concentration inks and: (**a**) Et:Etg solvent and 1-layer film surface; (**b**) IPE+triton-X solvent and 1-layer film surface; (**c**) Et:Etg solvent and 10-layer film surface; (**d**) IPE+triton-X solvent and 10-layer film surface; (**e**) Et:Etg solvent and 10-layer film cross-section; (**f**) IPE+triton-X solvent and 10-layer film cross-section.

We can also affect the microstructure by using high boiling point solvents and surfactants. In the present work, we used Et:Etg (50:50) solvent as a high boiling solvent and triton-X as a surfactant in combination with IPE. We have observed different microstructures for both cases, as shown in Figure 5. For both of the films based on Et:Etg and triton-X with IPE solvents, after printing one layer, the surface showed a substantial amount of flaking, as shown in Figure 5a,b; however, after the deposition of ten layers, as shown in Figure 5c,d the films appeared consolidated. Cross-section analysis (shown in Figure 5e,f) did not reveal substrate adhesion issues. However, in the case of the films based on Et:Etg inks, somewhat larger pores at the substrate interface, and thus smaller attachment areas, were observed.

Substrate roughness has an impact on film formation. To study the substrate’s role on microstructural variation, we used frosted glass having a relatively rough surface as a substrate for comparison.

**Figure 6:**
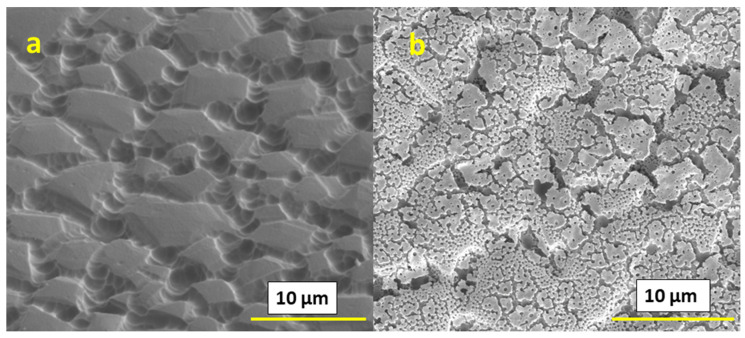
Surface SEM images of (**a**) bare frosted glass and (**b**) 0.4 M conc. 1-layer film on frosted glass.

Figure 6a shows the microstructure of bare frosted glass, and Figure 6b shows one layer of 0.4 M conc. ink deposited at a 300 °C printing temperature on frosted glass. We did not observe any significant differences for layer formation in this case, as compared to the films printed on a smooth glass surface. 

**Figure 7:**
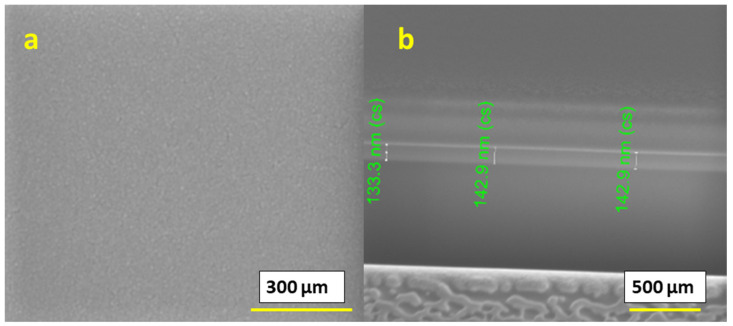
SEM images of 0.4 M ink 1-layer film printed at 60 °C; (**a**) surface image and (**b**) cross-section.

Furthermore, we studied films for a 0.4 M IPE-based solution printed at a 60 °C temperature and subsequently annealed at 400 °C. We observed that the surface of the obtained films was continuous, dense, smooth and homogeneous, as shown in Figure 7a. However, the thickness of a single-layer printed film was in the range of 130–145 nm, as shown in Figure 7b, and was comparatively thinner than that of the 300 °C printed films. The films did not contain any cracks or flaking. Such films can be suitable for applications that require dense films.

From all of the obtained results, we concluded that the observed mud cracks on some of the high-temperature deposited films might occur due to a dehydration phenomenon [20], which was not the case for low-temperature printed film. For films printed at a high temperature, porosity is likely related to the extremely rapid drying process and boiling of the solvent on the surface during drying, and it can be influenced by the solvent and its boiling point, as well as the use of surfactants. The thickness of the films can be varied by sequential repetition of the printing process, resulting in layer-by-layer growth. Additional variations in layer thickness can be introduced by finetuning the precursor concentration. Films printed at a 300 °C precursor concentration can be tuned within a small range (larger variations of this concentration result in substantial microstructural changes, as described above). Films printed at a 60 °C precursor concentration can be varied in a much broader range, allowing thickness to be finetuned with high precision. Depending on specific applications, one can use a set of parameters that include the printing temperature, ink concentration, ink solvent and substrate in order to obtain a suitable microstructure.

## 4. Conclusions

In summary, we demonstrated the fabrication of ceria films having a cubic CeO_2_ crystallographic phase by inkjet printing technique, where we could control the microstructure and thickness of the deposited films by tuning multiple printing parameters. Two printing temperature regimes, below and above the thermal decomposition temperature for the precursor, result in either a dense or porous microstructure that can be further tuned by varying parameters, such as the precursor concentration, solvent composition and solvent mixing ratios, use of surfactants, and substrate roughness. This parameter field provided enough degrees of freedom for one to obtain ceria films with a microstructure that is desirable for a broad range of applications. 

This fabrication approach has features of significant interest due to its low cost, its simplicity, a variety of controllable parameters, and its industrial scale-up viability. A substantial advantage of this technique is sustainable production. The drop-on-demand approach ensures that no material is wasted during deposition. The additive nature of inkjet printing and digital-patterning features eliminate the need for eventual lithography where patterned surfaces are needed. The low-energy consumption of these tools along with the absence of vacuum equipment further add to the sustainability of this fabrication approach.

## Figures and Tables

**Table 1:** Various inkjet printing parameters used in the present work.

Precursor	Ce(NO_3_)_3_·6H_2_O
Solvents for ink preparation	2-isopropoxy ethanol (IPE) Ethanol:Ethylene glycol (Et:Etg) 50:50 IPE with surfactant triton-X
Ink concentrations	0.2 M 0.4 M 0.6 M
Substrates for printing	Smooth glass Rough/frosted glass
Substrate cleaning steps prior to printing (20 min sonication in an ultrasonic bath for each step)	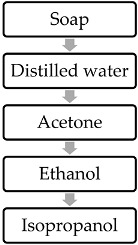
Inkjet printer model	Piezoelectric DOD inkjet printer Xaar with 80 pl printhead
Printing temperatures	60 °C 300 °C
Geometric surface area of printed films	0.9 cm × 1.5 cm
Annealing temperature and time	400 °C for 45 min

## Data Availability

The data reported in this study are available on request from the corresponding author.
